# What do we know about volumetric medical image interpretation?: a review of the basic science and medical image perception literatures

**DOI:** 10.1186/s41235-019-0171-6

**Published:** 2019-07-08

**Authors:** Lauren H. Williams, Trafton Drew

**Affiliations:** University of Utah, Salt Lake City, UT USA

**Keywords:** Medical image perception, Radiology, Visual search, Expertise, Volumetric medical images

## Abstract

Interpretation of volumetric medical images represents a rapidly growing proportion of the workload in radiology. However, relatively little is known about the strategies that best guide search behavior when looking for abnormalities in volumetric images. Although there is extensive literature on two-dimensional medical image perception, it is an open question whether the conclusions drawn from these images can be generalized to volumetric images. Importantly, volumetric images have distinct characteristics (e.g., scrolling through depth, smooth-pursuit eye-movements, motion onset cues, etc.) that should be considered in future research. In this manuscript, we will review the literature on medical image perception and discuss relevant findings from basic science that can be used to generate predictions about expertise in volumetric image interpretation. By better understanding search through volumetric images, we may be able to identify common sources of error, characterize the optimal strategies for searching through depth, or develop new training and assessment techniques for radiology residents.

## Significance

Volumetric medical images, such as computed tomography (CT) scans, consist of a series of stacked two-dimensional (2D) images, allowing for more accurate representation of the three-dimensional (3D) nature of the body’s anatomical structures. In recent years, there has been a steady increase in the number of volumetric medical images interpreted in diagnostic radiology. Although volumetric images are typically associated with better performance, missed or incorrect diagnoses remain prevalent in radiology. In this review, we will discuss findings from basic scientific research on visual attention and memory that may aid in our understanding of volumetric medical image search. In addition, we will discuss what is already known about volumetric image search through a review of the literature on medical image perception. Although there are currently substantial gaps in our knowledge of how best to search through volumetric images, this type of research might ultimately reveal superior search strategies for evaluating volumetric images, determine when errors are likely to occur, or lead to improved training methods for new radiologists.

## Introduction

**Fig. 1:**
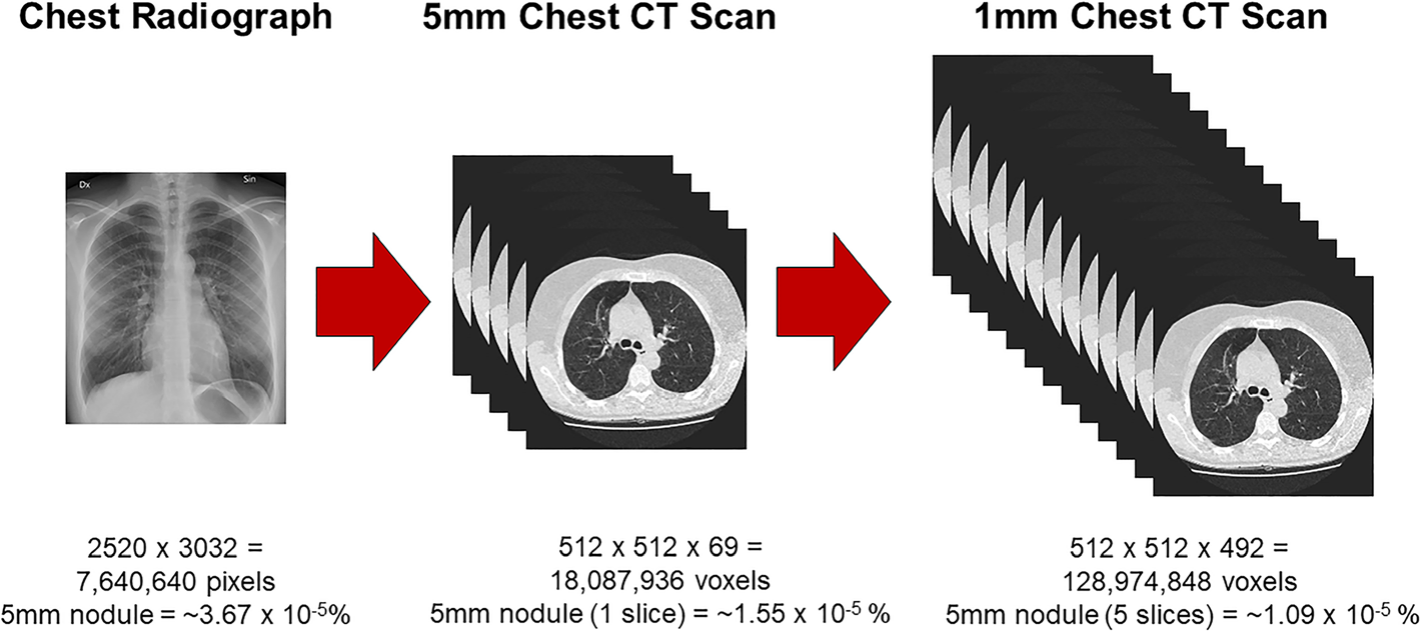
Size comparison of two-dimensional medical images and volumetric medical images. Image sizes are estimates and actual image sizes may vary considerably between cases. Lung nodule size estimates assume a 96-dpi monitor. CT, computed tomography

Volumetric medical imaging, such as CT, magnetic resonance imaging (MRI), or digital breast tomosynthesis (DBT), helps retain the 3D nature of the body’s internal structures by stacking multiple cross-sectional images. This imaging technique often results in a massive amount of information for the radiologist to evaluate (Andriole et al., 2011): a single chest radiograph is now often supplemented with a chest CT with a stack of 1000 high-resolution images (Fig. 1). Unfortunately, abnormalities are sometimes very small relative to the overall size of the image. To illustrate this point, Rubin (2015) calculated that lung cancer nodules between 4 mm and 10 mm in size make up 0.01% or less of the total volume in a typical chest CT scan. Lung cancer nodules of this size would only be visible on a handful of slices, rendering them undetectable for the vast majority of the radiologist’s overall search time (Rubin, 2015). How do expert radiologists efficiently sort through all of this information and detect potential abnormalities? Are there optimal strategies for navigating through volumetric images? Unfortunately, despite decades of medical image perception research, relatively little is known about expertise in the interpretation of volumetric medical images. However, given the increasing number of volumetric images in radiology, answering these questions will likely be at the forefront of medical image perception research in the coming years (McDonald et al., 2015).

The purpose of this manuscript is to review the literature and identify the current gaps in our understanding of volumetric image interpretation using a basic-science framework. First, we will discuss the merits of using basic scientific research on attention and memory to generate informed predictions about medical image perception. Next, we will discuss nine research areas that we feel best represent the current priorities of the field (Table 1). In each of these sections, we will discuss relevant findings from the basic science and medical image perception literatures and highlight promising areas for future research. This review should not be considered an exhaustive account of the literature. For example, the debate that surrounded the transition from analog to digital in radiology will not be covered in depth. Although the history of volumetric imaging is an interesting topic in its own right, it is beyond the scope of this review. In addition, we will not provide detailed discussion of the unique methodological challenges involved in volumetric imaging research and the approaches researchers have used to address them. Instead, we direct the reader to existing resources that cover this topic in depth (Rubin, Drew, & Williams, 2018; Venjakob & Mello-Thoms, 2015). Rather, this manuscript is a selected review of the literature on volumetric image perception through the lens of basic research on visual attention and memory. Although many of these topics undoubtedly pertain to 2D imaging as well, the primary intent of this manuscript is to focus on issues most relevant to volumetric imaging and serve as a catalyst for future research in this area.

**Table 1:**
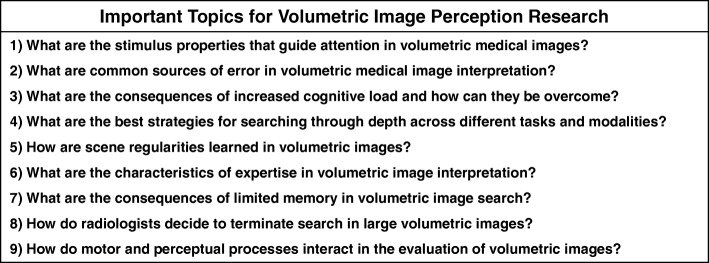
Important research areas for volumetric image perception

## What can we learn about medical image perception from basic scientific research?

**Fig. 2:**
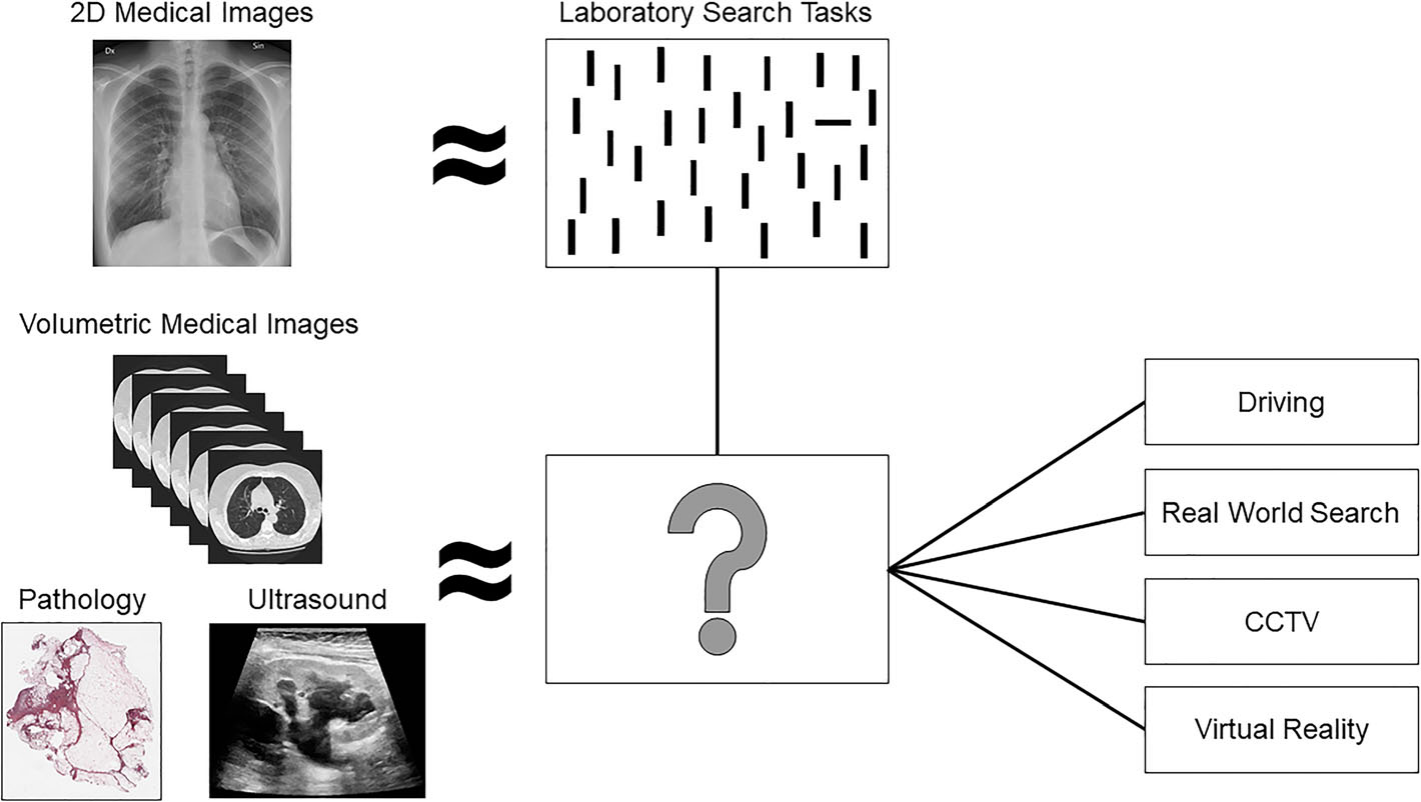
Although many findings from laboratory visual search tasks have been replicated in the medical image perception literature (e.g., Evans, Georgian-Smith, et al., 2013; Drew et al., 2013), there is no clear analog to volumetric images in the basic science literature. However, insight for future research directions on volumetric image search might be gained from findings on 2D visual search, as well as growing research in the realms of driving, real-world visual search, closed-circuit television (CCTV), and virtual reality. Ultrasound image reprinted from Hansen et al. (2016). Ultrasonography of the kidney: a pictorial review. *Diagnostics*, *6*(1), 2., and used here under the Creative Commons License. Pathology image obtained from National Cancer Institute Clinical Proteomic Tumor Analysis Consortium Sarcomas (CPTAC-SAR) collection 2018 and used here under the Creative Commons License

For several decades, researchers have sought to characterize how expert radiologists interpret medical images. Concurrently, cognitive scientists have been building a vast body of literature on visual search using tightly controlled laboratory tasks, such as “find the horizontal line amongst vertical lines.” At first glance, these artificial tasks seem to have little in common with complex radiology tasks, such as identifying signs of breast cancer in a mammogram. However, at their core, both of these tasks can be characterized as visual search and rely on the same mechanisms (Wolfe, Evans, Drew, Aizenman, & Josephs, 2016). In recent years, cognitive scientists have demonstrated the remarkable potential of applying findings from basic science to real-world tasks, such as radiology (Fig. 2). For example, observers in the laboratory often fail to notice a person walk through a basketball game wearing a gorilla suit when they perform a secondary task (e.g., counting the number of passes between players), a phenomenon known as “inattentional blindness” (Simons & Chabris, 1999). Similarly, 83% of radiologists missed a matchbook-sized gorilla image embedded into a slice of a chest CT scan when they were looking for signs of lung cancer (Drew, Võ, & Wolfe, 2013). This research may help explain why incidental findings, which are unexpected abnormalities that are not the primary focus of search, are sometimes missed in radiology (Wolfe, Soce, & Schill, 2017).

Examples of translational research from basic science to radiology, which have been thoroughly summarized elsewhere (Wolfe, 2016; Wolfe et al., 2016), highlight the promise of using our knowledge of human cognition to make predictions about how radiologists search through medical images and when they will be most susceptible to error. However, volumetric imaging has created a new set of challenges for both radiologists and the perception scientists seeking to better understand them. Volumetric imaging was first introduced to clinical practice in the 1970s, but recent years have seen a dramatic increase in the size and number of volumetric images being interpreted in the radiology reading room (Andriole et al., 2011; McDonald et al., 2015). For example, the number of cross-sectional images at one institution increased tenfold between 1990 and 2010 (McDonald et al., 2015). Unfortunately, the majority of research on medical image perception is based on 2D images, such as chest radiographs. In basic science, there is an extensive literature on visual search in 2D laboratory tasks and a growing literature on search in the 3D world. However, volumetric images do not fall neatly into either of these categories (Fig. 2). Nonetheless, there are a number of findings from these two bodies of literature that may provide insight on volumetric image interpretation, which we will highlight in this review.

Although much of a radiologist’s job can be characterized as decision-making, such as determining if a suspicious finding is cancerous or benign, this review will focus on how potential abnormalities are located and identified using visual search. To frame the discussion on visual search, we will primarily rely on the Guided Search model (Wolfe, Cave, & Franzel, 1989). The Guided Search model posits that early information guides attention in a bottom-up or top-down manner toward specific features in the scene. Bottom-up guidance is driven by the properties of the stimulus itself. For example, in the absence of another task, a bright red poppy in a field of daisies is likely to capture attention. In contrast, top-down guidance is driven by the observer’s internal state and selection history. Top-down attention can often override the effects of bottom-up mechanisms. For example, target representations held in memory can help guide attention away from salient distractors (e.g., the red poppy) and toward features in the environment that match the target’s features. Together, bottom-up and top-down factors generate a priority map that directs attention to areas in the scene that are more likely to contain the target.

### What are the stimulus properties that guide attention in volumetric medical images?

Bottom-up guidance in visual search can be highly effective when the most salient objects in the scene are consistent with your goals (e.g., identifying a large brain tumor), but harmful if your task involves detecting inconspicuous targets (e.g., small lung cancer nodules). Unfortunately, the most salient regions of medical images are not always the most informative for the radiologist. One well-established mechanism for limiting the influence of bottom-up information is through top-down knowledge about the task. All else being equal, experts should be able to better utilize a top-down strategy in medical image search than novices due to their extensive medical knowledge and past experience with similar images. For the same reason, the largest differences between experts and novices should be found in tasks that do not benefit from a bottom-up strategy. Broadly, these predictions have been well-supported in radiology, in addition to a number of other tasks and professions (Cooper, Gale, Darker, Toms, & Saada, 2009; Humphrey & Underwood, 2009; Koide, Kubo, Nishida, Shibata, & Ikeda, 2015; Lansdale, Underwood, & Davies, 2010). For example, novices’ eye-movements were closely predicted by a saliency map when analyzing single-slice brain CT scans for cerebrovascular incidents (Matsumoto et al., 2011, see also Nodine, Kundel, Lauver, & Toto, 1996). Similarly, experts viewed clinically relevant areas of low salience longer than novices. However, if clinically relevant areas were high-salience, experts and novices’ eye-movements did not differ (Matsumoto et al., 2011).

Future research is needed to determine the features that influence the detectability of abnormalities in volumetric medical images. In chest radiographs, researchers have used eye-tracking to make distinctions between lesion properties that capture attention initially during search (as measured by time to first hit) and those that hold attention once the abnormality is detected (as measured by dwell time) (Krupinski, Berger, Dallas, & Roehrig, 2003). In the context of guided search, “time to first hit” provides an index of the stimulus properties that more effectively guide attention to the lesion during visual search, whereas dwell time likely reflects recognition or decision-making processes. Although a number of characteristics (e.g., signal-to-noise ratio, conspicuity, location, and calcification) were evaluated, none of these features influenced how quickly attention would be directed to the relevant location in the image. However, both nodule size and conspicuity influenced dwell time on the lesion and predicted overall nodule detection rate. In contrast, Carmody, Nodine, and Kundel (1981) found that nodule conspicuity influenced both search and decision-making processes. Less conspicuous nodules were detected less often in a flash-viewing paradigm and were associated with more comparative scans to normal structures in the image during free viewing (defined as a fixation on the abnormality followed by a saccade and a refixation). In future work, it would be beneficial to evaluate the role of comparison scans for identifying different types of lesions in volumetric images. For example, the decision-making process for identifying a lung nodule might involve comparing how the abnormality’s appearance changes through depth relative to normal structures in the image (e.g., blood vessels).

In order to determine which stimulus features improve detectability in volumetric image search, it may be fruitful to lean on the basic science literature. According to Wolfe and Horowitz (2004), there are four guiding attributes that have been well-established by converging evidence in the literature: motion, color, orientation, and size. Although all of these features are undoubtedly important for detecting abnormalities in medical images, motion is an attribute that is uniquely applicable to volumetric images. In volumetric images, structures may appear to move along the 2D plane as the observer navigates through the depth of the image, which is thought to elicit smooth pursuit eye-movements as the observer tracks these structures through depth (Venjakob & Mello-Thoms, 2015). In addition, certain abnormalities, such as lung cancer nodules, appear to flicker in and out of view when scrolling through the depth of the image due to rapid changes in the structure’s diameter. This phenomenon may mimic abrupt motion onset cues, which are known to capture visual attention (Abrams & Christ, 2003; Girelli & Luck, 1997; Jonides & Yantis, 1988; Theeuwes et al., 1999). Furthermore, motion can serve as a filtering mechanism in visual search and strongly predicts where attention will be allocated in dynamic scenes (Kramer, Martin-Emerson, Larish, & Andersen, 1996; McLeod, Driver, Dienes, & Crisp, 1991; Mital et al., 2011). In addition, even if movement is not a defining feature of the target, observers learn frequent associations between targets and their movements and use this information to guide search (Scarince & Hout, 2018).

Although basic science suggests that motion cues serve as an effective form of guidance to a target, only a few studies have addressed this topic in medical image perception. For example, researchers found that artificially inducing motion cues into static images increased detection ability for both mammograms and chest radiographs (Andia et al., 2009). In addition, researchers tested the prediction that searching in smaller windows would be superior to searching in larger windows in volumetric images because it would increase the ability to detect motion cues using foveal vision (Venjakob, Marnitz, Phillips, & Mello-Thoms, 2016). Although there were no overall differences in accuracy between conditions, a smaller image size was associated with locating abnormalities more quickly. Finally, Nakashima et al. (2016) tested whether lung nodules are less likely to be detected early in the trial, when task-relevant motion onset cues (e.g., lung nodules) are likely obscured by simultaneous motion onset cues from task-irrelevant information (e.g., blood vessels). They found a significant effect of nodule location for novices, but not experts, which suggests that experts do not need to rely as heavily on these bottom-up signals for target detection. This is likely because experts have additional mechanisms, such as strong target representations and enhanced holistic processing, which also aid in the detection of abnormalities.

### What are common sources of error in volumetric medical image interpretation?

During visual search, target representations in memory are thought to guide attention in a top-down manner toward features in the environment that match the target’s features (Olivers & Eimer, 2011; Olivers, Meijer, & Theeuwes, 2006; Soto, Heinke, Humphreys, & Blanco, 2005). In typical laboratory paradigms, the observer searches for a single well-defined target that is either cued on each trial or remains the same throughout the experiment. However, searching for targets in more realistic circumstances where information about the target is degraded may be more challenging. Search performance is best when information about the target is precise (e.g., picture cues), and search is guided less effectively by imprecise (e.g., word cues) or categorical (e.g., cats versus Garfield) target cues (Hout & Goldinger, 2015; Wolfe, Horowitz, Kenner, Hyle, & Vasan, 2004). In addition, when multiple targets are present in an image (e.g., Garfield and Nermal), the second target is less likely to be detected after the first target is located (Berbaum et al., 1990; Cain & Mitroff, 2013). This phenomenon was originally termed “satisfaction of search” which suggested the error was caused by prematurely terminating search following the detection of the first target (Berbaum et al., 1990; Tuddenham, 1962). However, subsequent research has cast doubt on this explanation (Berbaum et al., 1991), and these errors are thought to have multiple causes (Cain, Adamo, & Mitroff, 2013). As a result, the term “subsequent search misses” has been proposed as a theory-neutral alternative (Cain & Mitroff, 2013). Unfortunately, a radiologist’s task often represents the worst-case scenario for target representations: identifying an unspecified number of poorly defined abnormalities.

Given these challenges, it is particularly important to consider how different imaging techniques might improve the radiologist’s ability to locate abnormalities. For example, 2D medical imaging forces the observer to view organs as overlapping structures, which can obscure findings and provide inaccurate spatial relationships between anatomical structures. In contrast, although volumetric imaging is not truly 3D, there is less need to mentally translate anatomical structures from their 2D representations to the 3D world. Non-overlapping structures, as well as the availability of motion cues, may improve the ability to detect abnormalities in volumetric images. Aside from breast cancer screening, direct comparisons between volumetric images and their two-dimensional counterparts are rare (Andersson et al., 2008; Ciatto et al., 2013; Gennaro et al., 2010; Gur et al., 2009; Michell et al., 2012; Rafferty et al., 2013; Spangler et al., 2011). However, studies that used this approach have demonstrated that volumetric images are associated with improved accuracy (Adamo et al., 2018; Aizenman et al., 2017; Alakhras et al., 2015; Blanchon et al., 2007; Mathie & Strickland, 1997; Seltzer et al., 1995). Critically, these accuracy differences are generally driven by both an increase in hit rate and a decrease in false alarms. However, volumetric imaging is also associated with a substantial cost: a large increase in search time and a decrease in overall coverage (Adamo et al., 2018; Aizenman et al., 2017; Lago et al., 2018).

It is important to note that although volumetric imaging appears to be superior to other imaging techniques, both inter-observer variability and overall error rates in radiology suggest there is substantial room for improvement. In addition, recent research demonstrates that volumetric imaging may not be universally advantageous (Lago et al., 2018). In a comparison between 3D breast tomosynthesis (DBT) and single-slice DBT, there were no differences in performance when readers were asked to identify masses. In contrast, 2D imaging was associated with better detection of microcalcifications. The researchers proposed that volumetric imaging leads to less image coverage and an increased reliance on para-foveal processing. Therefore, there is likely to be a cost of volumetric imaging when abnormalities cannot be readily detected in the periphery.

**Fig. 3:**
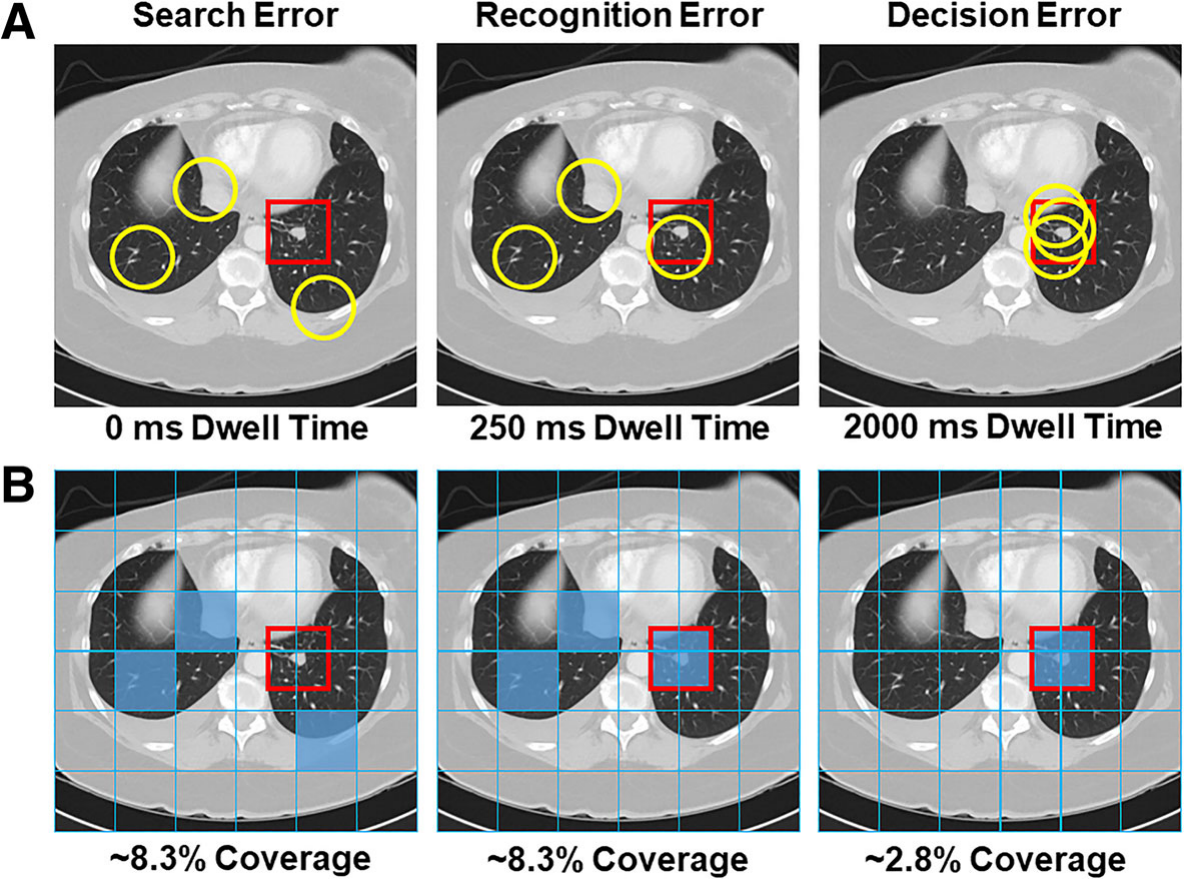
**a** Illustration of how a missed nodule can be classified as a search, recognition, or decision error using eye-tracking. Yellow circles represent fixations and the red square represents the region of interest for the abnormality. **b** Example of image coverage calculation

In order to better understand the sources of error in volumetric image interpretation, it is beneficial to move beyond behavioral data. For example, if an observer misses a lesion, it is often unclear whether they failed to find it or located it but decided it should not be reported. For this reason, eye-tracking has widely been used to determine why abnormalities are missed in various radiology tasks, such as lung cancer screening (Manning, Ethell, & Donovan, 2004). In general, both false positive and false negative decisions are associated with longer dwell time, which indicates that incorrect decisions are often associated with additional scrutiny (Kundel, Nodine, & Krupinski, 1989; Manning, Barker-Mill, Donovan, & Crawford, 2006). Eye-tracking has also been used to identify three distinct types of errors: search errors occur when a lesion is never foveated, recognition errors occur when a lesion is fixated on briefly (for < 1 s) but not reported, and a decision error occurs when a lesion is fixated on for a prolonged period of time (> 1 s) but not reported (Fig. 3a, Kundel, Nodine, & Carmody, 1978).

In lung cancer screening with chest radiographs, decision-making errors are the most common error type, followed by recognition and search errors (Donovan & Litchfield, 2013; Kundel et al., 1978). However, Drew, et al., 2013 observed a relatively small proportion of decision errors in lung cancer screening using chest CT scans. In fly-by 3D colonography, a virtual navigation through an endoluminal reconstruction of the colon, the majority of errors were identified as recognition errors and search errors were almost non-existent (Phillips et al., 2013). In addition, errors were evenly distributed between search and recognition errors in the identification of microcalcifications in DBT (Lago et al., 2018). However, the errors were primarily recognition errors for the identification of masses. These studies are good examples of extending current eye-tracking metrics to volumetric images, which allows direct comparisons between these modalities. By doing so, researchers have discovered that volumetric imaging may improve the ability to accurately identify an abnormality once it has been located across a variety of tasks, which may be a benefit of non-overlapping structures (Drew et al., 2013; Lago et al., 2018; Phillips et al., 2013). However, the distribution of errors can differ substantially based on the nature of the task or even between search strategies within same task (Drew et al., 2013; Lago et al., 2018). In future research, it will also be important to identify how the distribution of errors changes over the course of training in the interpretation of volumetric medical images, which may ultimately provide insight on the type of assistance (e.g., computer-aided detection) that would be most beneficial across levels of experience.

Although similar approaches have been used to classify errors in 2D and volumetric images (Drew et al., 2013; Lago et al., 2018; Phillips et al., 2013), it is largely unclear whether the thresholds for these categories are appropriate for volumetric images. It is also important for researchers to consider the appropriateness of applying these categories to different types of tasks. Certainly, an abnormality that is not fixated on indicates some level of search error, but determining whether fixational dwell time for an intermediate time (e.g., 500 ms) constitutes a recognition or decision error likely depends on both the task at hand and the level of expertise of the observer. For example, overall nodule dwell time in chest radiographs was lower for experts than trainees, which was mirrored by a shift to more recognition errors relative to decision-making errors (Donovan & Litchfield, 2013).

An alternative approach to Kundel’s classic error categorization was recently advanced by Cain et al. (2013). After recording eye movements for thousands of trials, they used a data-driven approach for the task in question (a multiple-target, visual search task in their case) to describe different types of errors. Data-driven approaches allow the threshold between recognition and decision errors to be adjusted for a given stimulus based on the distribution of dwell times or the average search slope. Using this approach, Cain et al. (2013) identified a threshold ~ 25% of the value typically used as a threshold in medical image perception. Notably, there was little evidence to support a clear, qualitative distinction between recognition and decision errors. Rather, the data could be more adequately described by models of perceptual decision-making, such as drift diffusion (Ratcliff & McKoon, 2008), that posit that evidence is slowly accumulated during the fixation on an item until a decision threshold is reached. From this perspective, recognition and decision errors occur on a continuum rather than as distinct categories. In addition, Cain et al. (2013) demonstrated that search errors for the second target could be further sub-divided into novel categories. On some trials, the search was terminated as soon as the first target was identified with no attempt to locate the second target (“strategy” error). On other trials, the first target was re-fixated on during search (“resource depletion” error), which suggests working memory resources might have been depleted by maintaining information about the first target (Cain and Mitroff, 2013). This research highlights that there is not a one-size-fits-all approach to error classification between tasks, as well as the potential for data-driven classification to provide additional insight on sources of error in visual search. Although this approach requires a large amount of data, which can be difficult to collect with radiology observers, it may be informative to use a data-driven method to create a taxonomy of errors in volumetric image search. For example, an abnormality might be missed in a volumetric image if the abnormality is visible during search but never fixated on, but a miss error could also occur if the slice of the image that contains the abnormality is never visited. Although these would both be considered search errors under Kundel’s classification system, these likely represent different sources of error.

### What are the consequences of increased cognitive load and how can they be overcome?

In light of the increased ability to detect abnormalities in volumetric images, one might expect volumetric images to be associated with a reduced cognitive load. However, medical students report greater mental effort when viewing volumetric images, which may be due to the increased size, complexity, and evaluation time associated with these images (Stuijfzand et al., 2016). This finding appears to be supported by pupil size, a physiological measure of cognitive load (Porter, Troscianko, & Gilchrist, 2007; Unsworth & Robison, 2018), which increases with search time in volumetric images (Stuijfzand et al., 2016). Along similar lines, recent work with breast pathologists examining digital pathology slides has found that pupil diameter is sensitive to perceived case difficulty: more difficult cases were generally associated with a larger pupil diameter (Brunyé et al., 2016). Findings from a wide variety of sources suggest that visual search is impaired when working memory is taxed. Concurrent spatial working memory load reduces the efficiency of visual search in both laboratory and applied tasks, such as driving (Oh & Kim, 2004; Recarte & Nunes, 2003). In addition, salient bottom-up features are known to capture attention more effectively under cognitive load (Matsukura, Brockmole, Boot, & Henderson, 2011). Typically, observers in natural tasks seek to minimize their cognitive load by frequently scanning their environment, particularly when memory load is high and the task is unpredictable (Droll & Hayhoe, 2007). This effect seems to be exaggerated in novices: weaker chess players favor moves that will reduce working memory load, such as decreasing the number of pieces on the board (Leone, Slezak, Cecchi, & Sigman, 2014).

In radiology, increases in cognitive load and fatigue may have a detrimental impact on patient care. Discrepancies increase during the final hours of a long work day, and volumetric images have been identified as a risk-factor for these discrepancies (Ruutiainen, Durand, Scanlon, & Itri, 2013). After viewing CT images, observers have reduced accuracy, greater visual fatigue, and increased visual strain (Krupinski et al., 2012). Similarly, think-aloud protocols reveal that radiologists verbalize more often about efficient search strategies and image manipulation skills in volumetric images than in 2D images (van der Gijp et al., 2015). Furthermore, one study suggests that residents are more affected by fatigue than experts while detecting abnormalities in abdominal CT (Bertram et al., 2016). In future research, it will be necessary to determine which search strategies best offset the cognitive load associated with volumetric medical images.

### What are the best strategies for searching through depth across different tasks and modalities?

**Fig. 4:**
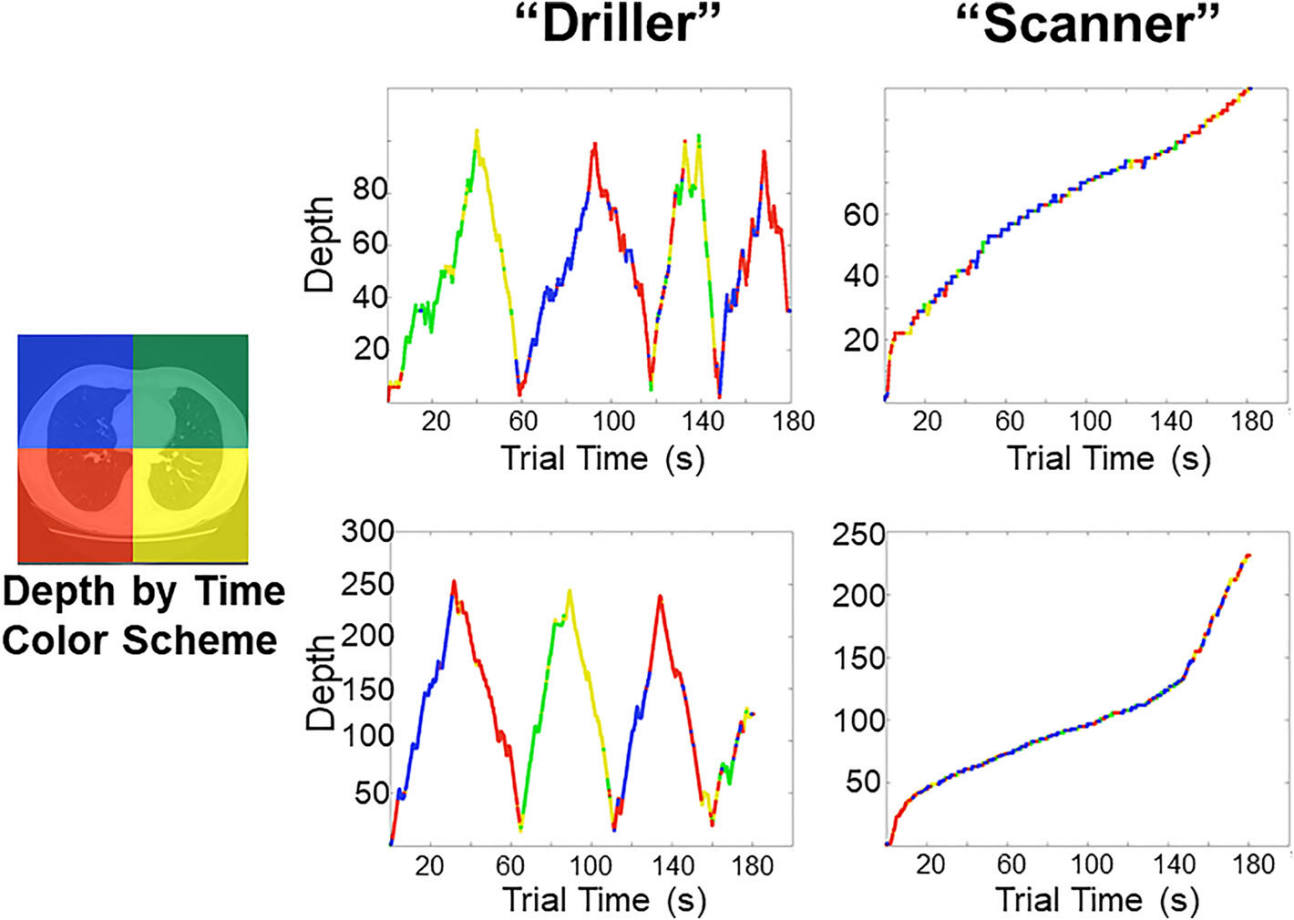
Two strategies emerge when searching through chest computed tomography (CT) scans for lung nodules: scanning and drilling. Scanners move their eyes along the two-dimensional plane while slowly scrolling through depth. In contrast, Drillers keep their eyes relatively stationary in one region at a time while rapidly scrolling through depth. At present, it is largely unknown how these strategies translate to other modalities or tasks. Figure reprinted with permission from Rubin et al. (2018). Perception of volumetric data. In *Handbook of medical image perception & technology* (Vol. 2). Cambridge, United Kingdom: Cambridge University Press. Original figure was recreated from Drew, Võ, Olwal, et al. (2013). Scanners and drillers: characterizing expert visual search through volumetric images. *Journal of Vision*, *13*(10), 3

One particularly promising avenue of research is to explore how variation in scrolling behavior might relate to search performance. Drew et al. (2013) found that adopting a strategy of rapidly “drilling” through depth while maintaining fixation was superior to “scanning” the x and y plane while slowly moving through depth during lung cancer screening (Fig. 4). Although this study did not test the use of motion cues directly, it is possible that drilling allows the observer to more effectively take advantage of transient motion cues to distinguish blood vessels from nodules that appear to “pop in and out of view” while scrolling through depth. In support of this proposal, Wen et al. (2016) found that scanners and drillers make use of different bottom-up cues in lung cancer screening tasks. It appears that drillers are better able to make use of salient motion cues, whereas scanners’ search behavior is driven largely by 2D saliency. In addition to overall performance differences, the distribution of errors differed between scanners and drillers: drillers tended to have more recognition errors than scanners, and scanners tended to make more search errors than drillers. At present, it is unclear how these search strategies emerge over the course of training. Drew et al. (2013) found that drillers tended to read more CT cases per week than scanners. However, there were no differences in search strategy based on years of experience, and the sample size was not large enough to fully tease apart the effects of search strategy versus experience in relation to overall performance.

In future research, it will be beneficial to investigate the best search strategies in volumetric images across different tasks and modalities. Search strategies that are most effective for a given task (e.g., detecting focal abnormalities, such as lung cancer nodules) may not be optimal for abnormalities defined by different bottom-up properties (e.g., detecting diffuse abnormalities, such as pneumonia). There are some good examples of these comparisons from studies using 2D medical images (e.g., Gegenfurtner & Seppänen, 2013; Krupinski, 2005; Krupinski et al., 2003; Mousa et al., 2014). For example, when viewing chest radiographs, different search patterns are elicited for diffuse abnormalities, focal abnormalities, and normal images (Kok, De Bruin, Robben, & van Merriënboer, 2012). Furthermore, experts and novices have different patterns of behavior based on the type of abnormality. Diffuse abnormalities generally led to shorter and more dispersed fixations, but this effect was more pronounced in the students. In contrast, focal abnormalities were characterized by longer fixations at a given location. Notably, in a direct comparison between breast tomosynthesis and chest CT, Aizenman et al. (2017) found that breast tomosynthesis led to a more rigorous drilling strategy than chest CT. Furthermore, no observers adopted a scanning strategy. Similarly, although both scanners and drillers could be identified in examinations of abdominal and pelvic CT, there was no accuracy advantage associated with being a driller (Kelahan et al., 20192019). The scanner/driller distinction has also recently been extended to the realm of digital pathology, where clinicians pan and zoom into large images to more closely view suspicious regions (Mercan, Shapiro, Brunyé, Weaver, & Elmore, 2018). In this domain, scanning appears to be the dominant strategy, but there were no differences in performance. In future research, it may be beneficial to evaluate the stimulus properties that influence the relative proportion of these strategies in volumetric images in a more systematic manner.

A recent study approached the question of inter-observer variability in scan patterns using a novel tool called ScanMatch (Crowe, Gilchrist, & Kent, 2018). The ScanMatch method compares fixation sequences across observers by assigning a letter value to each region and generating a string sequence for each participant. These strings are then compared between observers and a similarity score is obtained. In this study, observers viewed two runs of a fixed speed presentation of brain MRI scans. Overall, experts engaged in more similar scan patterns than novices. In addition, greater similarity was associated with better performance. These results could be explained in a number of ways. First, it is possible that experts are driven by statistical irregularities picked up in the first presentation of the stimulus, which is supported by increased similarity scores for true positives and lower similarity scores for false negatives. However, the same pattern was not found for true negatives, which led the authors to suggest that experts might instead use more systematic search strategies in the absence of statistical irregularities. Consistent with this view, observers adopt endogenous systematic search strategies in visual displays that are lacking in features that typically guide search behavior, such as saliency and semantic information (Solman & Kingstone, 2015). In addition, systematic search strategies were more closely associated with the strongest performers. In future research, it would be interesting to test these predictions more directly in volumetric image search, particularly as it relates to the reliability of scrolling behavior through depth using more clinically-valid free-scroll paradigms.

### How are scene regularities learned in volumetric images?

In a typical laboratory search task, the observer might be asked to indicate whether a target is present or absent in a display that consists of randomly ordered objects on a blank background. In contrast, real-world scenes are rich with context, and neighboring objects are often closely related to each other. A toothbrush near the bathroom sink will be identified more quickly than a toothbrush placed on a piano or floating in midair (Torralba, Oliva, Castelhano, & Henderson, 2006). This form of top-down guidance is referred to as scene grammar (Võ & Wolfe, 2015). To a knowledgeable observer, medical images are also highly structured and contextual. For example, gallstones always occur in the gallbladder, which is situated under the liver. This greatly constrains the regions of an abdominal CT scan that need to be evaluated for gallstones. This type of top-down knowledge is thought to alter the areas of chest radiographs that are attended over the course of training, leading to qualitatively different search patterns between experts and novices (Kundel & La Follette, Jr., 1972; Manning, Ethell, Donovan, & Crawford, 2006). Importantly, this effect seems to develop organically without any explicit instructions on how to search through chest radiographs, which suggests it is strongly driven by top-down knowledge about where abnormalities are likely to occur rather than training on specific search strategies.

Although our knowledge of the world allows us to make an educated guess about where to find a toothbrush in a stranger’s house, we will likely find a toothbrush more quickly in our own bathroom due to repeated experience. In the laboratory, the response time benefit from repeated exposures to the same search array is referred to as contextual cueing (Chun & Jiang, 1998). Although these effects are typically observed in highly artificial search tasks, contextual cueing is also found in dynamic tasks where targets and distractors repeatedly move with a certain trajectory, 3D depth displays, outdoor environments, and virtual apartments (Chun & Jiang, 1999; Jiang, Won, Swallow, & Mussack, 2014; Kit et al., 2014; Li, Aivar, Kit, Tong, & Hayhoe, 2016; Zang, Shi, Müller, & Conci, 2017). Furthermore, although object-based information is a strong contextual cue (Koehler & Eckstein, 2017), contextual guidance does not necessarily depend on objects in a scene; this information can be extracted from statistical regularities in low-level visual features (Torralba et al., 2006). In addition, contextual cueing is tolerant to a number of changes between exposures (Song & Jiang, 2005).

Given the large size of volumetric medical images (Andriole et al., 2011; McDonald et al., 2015), it is undoubtedly important for radiologists to lean on some of the aforementioned mechanisms to narrow the search area down to relevant regions of space. It is simply not practical to search every pixel of a large CT scan (Fig. 1) and it is likely this top-down guidance is one of the biggest advantages of expertise (for reviews, see Gegenfurtner et al., 2011 and van der Gijp et al., 2016). However, unlike 2D medical image interpretation, the influence of top-down knowledge on the observer’s search strategy over the course of training when reading volumetric images is largely unknown, particularly as it relates to scrolling through depth. Typically, expertise studies approach these questions by analyzing indirect measures, such as image coverage or time to first hit, across levels of experience (e.g., Donovan & Litchfield, 2013; Manning et al., 2006). However, it can be difficult to disentangle the influence of medical knowledge versus learned statistical regularities using these indirect measures of top-down processing. A complementary approach to these indirect measures might be to train novice observers on artificial volumetric displays and determine how search behavior changes with experience.

### What are the characteristics of expertise in volumetric image interpretation?

The advantage of regularities in our environment is that we can form detailed scene representations, known as schemas, to guide visual search behavior. For example, contextual cueing appears to rely on spatial working memory resources for the expression, but not acquisition, of learned displays (Annac et al., 2013; Manginelli, Langer, Klose, & Pollmann, 2013). It is thought that spatial working memory rapidly links the current search configuration to schemas held in long-term memory, making the observer sensitive to statistical irregularities in their environment. In fact, familiar scenes presented for a fraction of a second can be accurately categorized (Potter, 1975), guide subsequent eye-movements (Castelhano & Henderson, 2007), and increase the detectability of novel objects in the scene (Brockmole & Henderson, 2005; Chen & Zelinsky, 2006). This phenomenon is referred to in the literature as “gist”, “holistic”, or “global” processing, and it is frequently studied using a flash moving-window paradigm (Castelhano & Henderson, 2007). In this paradigm, observers are shown a brief preview of the scene followed by a mask and a subsequent target cue. The search task is performed using a gaze contingent window, which eliminates the influence of online parafoveal processing and isolates the effect of scene preview (i.e., the initial holistic impression) on search behavior.

In radiology, the beneficial effects of scene preview appear to be more modest than those observed in the visual search literature. Scene previews before a lung cancer detection task were associated with small improvements in search time and fewer overall fixations (Litchfield & Donovan, 2016). However, these benefits did not correspond with an increase in accuracy and were only weakly associated with expertise. Furthermore, scene previews appeared to be harmful if the pathology varied between trials. Nonetheless, there is strong evidence that radiologists are able to rapidly detect statistical anomalies in medical images. Kundel and Nodine (1975) found that 70% of lung nodules were detected after chest radiographs were viewed for only 200 ms. Similarly, research has shown that mammographers can classify images as normal or abnormal at a rate above chance after viewing them for only 250 ms (Evans, Georgian-Smith, Tambouret, Birdwell, & Wolfe, 2013). However, the ability to localize these lesions was at chance (though see Carrigan, Wardle, & Rich, 2018). In addition, the majority (57%) of breast cancers and a large portion (33%) of lung cancers are fixated on in the first second of viewing, which is simply not enough time to perform a thorough search (Donovan & Litchfield 2013; Kundel, Nodine, Conant, & Weinstein, 2007; Kundel, Nodine, Krupinski, & Mello-Thoms, 2008). In addition, eye-tracking demonstrates that expertise is associated with substantial differences in search behavior: experts exhibit more circumferential scan patterns, shorter time to first fixation, greater fixation duration, a smaller fixation count, less image coverage, and reduced variability in gaze (Kundel & La Follette, Jr., 1972; McLaughlin, Bond, Hughes, McConnell, & McFadden, 2017). Notably, expert-like scan patterns may pre-date expert decision-making (Kelly, Rainford, Darcy, Kavanagh, & Toomey, 2016).

These findings have led to a series of models on medical image perception, which all feature holistic processing as a prominent component of expertise (Drew et al., 2013; Nodine & Kundel, 1987; Swennson, 1980). Swennson proposed a two-stage model. The first stage involves a pre-attentional filter, similar to feature integration theory (FIT), which rapidly selects certain areas of the image for processing. In the second stage, the areas marked during the first stage receive further scrutiny. Similarly, Nodine and Kundel (1987) proposed a global-focal search model. During an initial global impression, the image is rapidly compared to the observer’s schema of a normal image. In the next stage, perturbations between the image and the mental representation are further evaluated using focused attention. Finally, Drew et al. (2013) outlined a model that relies on two parallel pathways (see also Wolfe, Võ, Evans, & Greene, 2011). The nonselective pathway extracts global information from the image using a large field of view. The selective pathway extracts detailed visual information that supports object recognition using a more focal search. Although these models are nuanced, they all emphasize the importance of rapidly extracting global information to guide search behavior: an ability which is thought to increase with experience.

Although much is known about expertise in 2D images, there is far less research on expertise in volumetric images. Of the existing research, several rudimentary findings have been replicated in volumetric images (Table 2). For example, experts are more accurate, search faster, locate abnormalities more quickly, and exhibit more fixations in regions of interest (Bertram, Helle, Kaakinen, & Svedstrom, 2013; Cooper et al., 2009, 2010; Mallett et al., 2014). However, many findings based on 2D medical images have not been replicated using volumetric images (Table 2). For example, Bertram et al. (2013) found no differences in average fixation duration between experts and novices, which is typically used as an index of increased processing ability with expertise. In addition, the researchers found no group differences in saccadic amplitude, which is a key index of global processing ability. Similarly, Mallett et al. (2014) failed to find any differences in eye-movements between experts and novices in fly-by endoluminal CT colonography, aside from reduced time to first pursuit. However, both of these studies utilized tasks (e.g., enlarged lymph nodes, visceral abnormalities, and colon polyps) that are far removed from the tasks typically used in studies with 2D images. At present, it is unclear if these differences are due to the nature of the task or fundamental differences in how expertise is expressed in volumetric imaging. In fact, there are very few direct eye-tracking comparisons between 2D and volumetric search. In a rare example of this approach, Aizenman et al. (2017) found that breast tomosynthesis was associated with longer fixations and less image coverage than traditional mammography. However, saccadic amplitude was equivalent, which suggests an equal ability to rely on parafoveal processing in both modalities.

**Table 2:**
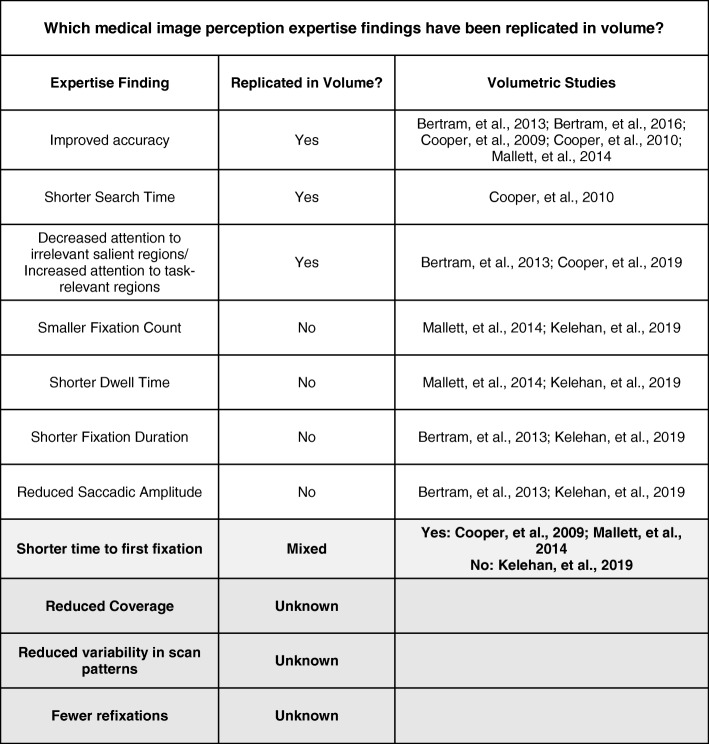
A list of common expertise-related findings in two-dimensional images. Many of these basic expertise findings have either not replicated or not yet been tested in volumetric images (particularly using free-scroll paradigms with stacked images)

The increase in holistic processing ability with expertise is one of the most important discoveries in the medical image perception literature, but it is almost completely unknown how these abilities might manifest in volumetric images. One possibility is that global impressions are continually formed on the 2D plane as the observer scrolls through depth. For example, the observer might fixate more quickly on abnormalities from the moment they are first visible on the screen (e.g., Helbren et al., 2014, 2015). If this is the case, analogs of eye-tracking measures associated with global processing in 2D image interpretation should transfer to volumetric images (Table 2). However, a global representation of the scene could also be formed by rapidly scrolling through the depth of the image prior to utilizing a more focal search pattern. There is support for this proposal in the literature. In real-world tasks, such as making a sandwich, observers conduct an initial scan of the scene, which helps them locate target objects more quickly during the task (Hayhoe, Shrivastava, Mruczek, & Pelz, 2003). Moreover, it is possible that global processing ability is expressed differently based on the search strategy of the observer. For scanners, global impressions might be established on the 2D plane with each transition through depth. In contrast, drillers might establish a global impression by scrolling through depth and then returning to layers of depth that were statistically anomalous.

In addition to scanners and drillers, other metrics of scrolling behaviors through depth have been proposed in relation to global processing ability (Table 3): the number of visits per slice, the number of oscillations (scrolling back and forth through less than 25% of depth), the number of half runs (scrolling back and forth through 25–50% of depth), and the number of full runs (scrolling back and forth through > 50% of depth) (Venjakob, Marnitz, Mahler, Sechelmann, & Roetting, 2012). Radiologists who engage in more full runs are thought to use a more global search process, which should increase with the experience of the observer. However, this proposal has not yet been tested, and these measures have not been widely used outside of this initial exploratory study using cranial CT images.

**Table 3:**
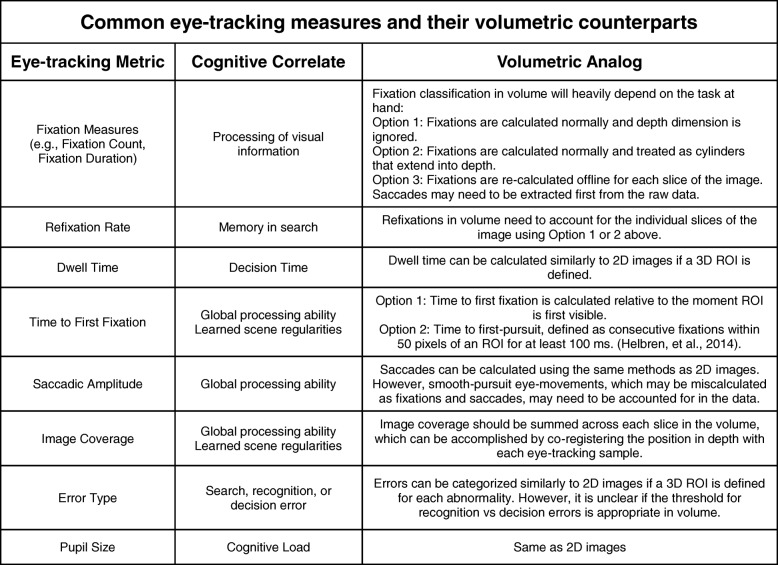
Common eye-tracking metrics, their cognitive correlates, and proposed analogs for volumetric medical images. ROI, region of interest

In other realms of medical imaging that might be considered similar to volumetric images, such as virtual microscopy, there is a clear link between expertise and global processing ability (Krupinski, Graham, & Weinstein, 2013; Krupinski et al., 2006). Although pathologists at all levels of experience were likely to select informative areas for the locations they would like to magnify, more experienced pathologists spent less time evaluating regions that ultimately would not be selected for magnification. This evidence, in addition to a number of critical behavioral and eye-tracking measures, suggests that experienced pathologists have an increased ability to rapidly extract the most important information from medical images. Other studies have highlighted the similarities in search strategies between digital pathology and volumetric imaging (Mercan et al., 2018), but it is largely unclear how these findings relate to expertise in either domain. In addition, there are clear differences between these images: choosing to view a visible part of the image at a greater resolution is not the same as scrolling to reveal visual information that is embedded throughout the depth of the image. We believe there are a number of promising areas for future research related to how search behaviors might differ in relation to expertise across a wide variety of areas (e.g., pathology, ultrasonography).

When discussing changes in search strategy with expertise, an important caveat should be considered: differences in search patterns between experts and novices do not necessarily mean that training the novice to use those strategies will improve performance. In many cases, the research indicates that strengthening the target template through greater exposure to examples of normal and abnormal images would be far more beneficial to the novice than instructing them where to look (Chen et al., 2017; Donovan & Litchfield, 2013; Kundel & La Follette, Jr., 1972; Manning et al., 2004; Nodine et al., 1996, 1999). Although it is tempting to identify shortcuts to expertise, most efforts to train novices to utilize new strategies or to follow the scan paths of experts have had modest success or limited generalizability (Gegenfurtner, Lehtinen, Jarodska, & Saljo, 2017; Kok et al., 2016; Litchfield, Ball, Donovan, Manning, & Crawford, 2010; Mello-Thoms, 2008; van Geel et al., 2017). When considering this issue, it may be helpful to consider which aspects of visual search might be enhanced by using these techniques. Training novices to mimic the search behavior of experts might improve overall search strategy, but it is doubtful these methods would substantially improve global processing ability, which is considered a hallmark of expertise. Rather, global processing ability is attributed to a greater ability to rapidly detect statistical abnormalities in an image via strong mental representations, which is acquired through extensive experience. Notably, expert radiologists search different areas of the image on each case, which is thought to be driven by the global properties of each image (Manning, Ethell, & Crawford, 2003). However, trained radiographers tend to skip the same regions consistently, which likely reflects a strategy more influenced by the prior probabilities of encountering an abnormality at a given location (Manning et al., 2003). These findings suggest that although both experts and novices rely on their previous experiences to guide search, experts have stronger mental representations to rely on than novices. Future research that seeks better training techniques should consider which elements of expertise require experience and which are learned strategies. In addition, it may be beneficial to focus on supporting radiologists at different stages of development rather than seeking shortcuts between them.

In addition to group-level differences between experts and novices, it may be equally beneficial to explore how idiosyncrasies in eye-movements relate to the substantial variability in performance observed among experts. Hayes and Henderson (2017) found that variations in scan patterns explain a large portion of the variance in individuals’ working memory capacity, speed of processing, and intelligence. Moreover, individual differences in scan patterns seem to be fairly stable across different types of tasks, even when adopting a rigid scan pattern may not be optimal (Andrews & Coppola, 1999; Henderson & Luke, 2014; Mehoudar, Arizpe, Baker, & Yovel, 2014; Paeye & Madelain, 2014; Poynter, Barber, Inman, & Wiggins, 2013; Rayner, Li, Williams, Cave, & Well, 2007). In addition, scan patterns reveal a great deal about an individual’s search strategy, such as a preference for speed or accuracy (Hogeboom & van Leeuwen, 1997). Many researchers have attempted to determine if there are domain general cognitive abilities associated with expertise in radiology, which may help predict who might become a better radiologist or explain why equal experience does not lead to equivalent performance. These approaches have largely been unsuccessful and paint a compelling picture of domain specificity with expertise (Beck, Martin, Smitherman, & Gaschen, 2013; Evans et al., 2011; Kelly, Rainford, McEntee, & Kavanagh, 2017; Leong et al., 2014; Myles-Worsley, Johnston, & Simons, 1988; Nodine & Krupinski, 1998). However, performance on the first trial of a visual search task predicts which individuals will perform well with experience, which suggests there may be important individual characteristics that have been overlooked in previous research (Ericson, Kravitz, & Mitroff, 2017). Research from the basic science literature suggests that differences in eye-movements may provide insight on these questions, but this has not yet been evaluated in the literature.

### What are the consequences of limited memory in volumetric image search?

Guided search posits that attention will be directed to the subset of items in your environment that are more likely to be your target. For example, if you are searching for romaine lettuce in the grocery store, attending to green items reduces the overall number of items that need to be evaluated. However, it stands to reason that visual search would be most efficient if the cognitive system kept track of which green items have already been evaluated in order to guide attention to novel locations and minimize unintentional eye-movements to previously visited locations. Such a mechanism would be particularly advantageous for professional visual searchers, such as radiologists, who need to efficiently determine which areas of large volumetric images they have already evaluated and when it is time to move on to another task. Many models of visual search carry the implicit assumption that previously attended objects will never be reevaluated (e.g., Treisman & Gelade, 1980). Consistent with this assumption, research has shown that observers search as if they have implicit memory about where they have recently looked: saccades are more likely to move in the same direction as the preceding saccade than the opposite direction (Klein & MacInnes, 1999), saccadic latency is higher to previously visited locations than to novel locations (Vaughan, 1984), and refixation rate more closely resembles a model that assumes memory of previous fixations than one that does not (Bays & Husain, 2012; Peterson, Kramer, Wang, Irwin, & McCarley, 2001).

A commonly proposed mechanism for this phenomenon is inhibition of return (IOR), which is a term used to describe delayed response times to probes in recently attended locations relative to novel locations (Posner & Cohen, 1984). In real-world search tasks, IOR is thought to serve as a foraging facilitator (Klein & MacInnes, 1999). In support of this hypothesis, Klein and MacInnes (1999) found that saccades to a probe in a *Where’s Waldo* search task were delayed in recently (2–3 back) fixated locations. However, despite the obvious utility of a memory mechanism in visual search, evidence for it has been surprisingly mixed. Horowitz and Wolfe (1998) found that search efficiency was not affected when objects moved around in the scene every 100 ms, which suggests that memory typically plays little to no role in visual search. This extreme model of a memoryless search has been challenged many times (e.g., Geyer, Von Mühlenen, & Müller, 2007; Kristjánsson, 2000; Peterson et al., 2001; Shore & Klein, 2000), but these results do indicate that visual search may involve less memory for previously visited locations than our intuition suggests.

One proposal that attempts to reconcile these conflicting pieces of evidence is that IOR serves to discourage perseveration in visual search, but is too limited in capacity (~ 4 items) and takes too long to develop (~ 200–300 ms) to produce a search that “samples without replacement” (Wolfe, 2003). These limitations also cast doubt on the idea that IOR might play a substantial role when scrolling through large, volumetric medical images that necessitate hundreds of fixations. Furthermore, IOR appears to be severely disrupted by interruptions, particularly when the search array is no longer visible (Takeda & Yagi, 2000). This suggests that IOR may be closely tied to objects in the scene rather than spatial location. If IOR is only effective when tagged objects are visible, moving to new layers of depth may disrupt the process and further limit the utility of an IOR mechanism in volumetric image search.

It appears that implicit memory for previously viewed locations is fairly limited, but what about explicit memory? When searching a complex scene (e.g., *Where’s Waldo*) observers are able to distinguish their own eye-movements from randomly generated scan paths (Foulsham and Kingstone, 2013a, 2013b; Võ, Aizenman, & Wolfe, 2016). However, observers are close to chance at distinguishing their own fixations from a stranger’s fixations, particularly in static displays (Foulsham and Kingstone, 2013a, 2013b; van Wermeskerken, Litchfield, & van Gog, 2018; Võ et al., 2016). One explanation for this pattern of results is that observers rely on their knowledge of where it would make the most sense to look in an image to perform the task rather than maintain a representation of their scan path in memory (Foulsham & Kingstone, 2013a, 2013b; Võ et al., 2016). In further support of this view, observers are able to better discriminate their own eye-movements in a given scene when the second observer searched for a different item and over-represent the likelihood that objects that are easily accessible in memory were fixated on during visual search (Clarke, Mahon, Irvine, & Hunt, 2017). Together, these results point to surprisingly poor explicit memory for previously visited locations, which is primarily driven by informed guesses about where someone should have looked in a scene rather than memory per se.

What implications do poor implicit and explicit memory have for radiologists searching through volumetric images? If you forget where you have searched for your keys in the morning, the worst-case scenario is that you are a few minutes late for work because you checked the same places more than once. However, it would be highly consequential for a radiologist to forget whether or not they have checked everywhere for signs of trauma after a car accident. This may be particularly relevant for volumetric images: it may be more difficult to maintain a representation of where you have already searched when images increase in size. Furthermore, it is more time consuming to start over or retrace your steps in a large CT scan than in a radiograph if you lose your place, which is even more consequential in light of the increase in radiologists’ workload due to volumetric imaging (Andriole et al., 2011; McDonald et al., 2015).

The visual search literature suggests that knowledge of where you have already searched is largely based on statistical regularities and scene context (Chun & Jiang, 1998; Clarke et al., 2017; Torralba et al., 2006). There is little reason to suspect that expert radiologists would differ in this finding, but there are clear limitations to this strategy. For example, following an interruption, where does the radiologist choose to resume their search? One possibility is that radiologists have forgotten where they have already searched and unknowingly revisit those locations, which could be observed by tracking the number of refixations and the accuracy of search resumption following the interruption (Williams & Drew, 2017). However, from these measures alone, it is unclear if areas are revisited because they are forgotten or if they are consciously revisiting these areas in order to better recall what they intended to do next. This limitation highlights the need for more direct measures of memory in applied visual search tasks. If explicit recall is largely based on knowledge of which areas should be searched rather than knowledge of where you have actually searched, this strategy may lead to inaccurate search resumption following an interruption. One possibility is that relevant areas will be prioritized and more resistant to the effects of interruptions. Alternatively, if memory recall is primarily based on which areas should be searched, the most relevant structures might be recalled despite the fact that they were never searched. One way to disentangle these possibilities would be to combine indirect measures (e.g., eye-tracking) with more direct measures (e.g., periodic probes) to determine which areas are more likely to be reported as searched following an interruption.

Consistent with a poor memory account, radiologists often search a surprisingly small portion of medical images, even though coverage is negatively associated with error rate within an expert population (Drew et al., 2013; Rubin et al., 2015; Thomas & Lansdown, 1963). For example, Drew et al. (2013) found that only 69% of the lung was searched during lung cancer screening using a 5° useful field of view (UFOV) estimate. Drillers covered more of the image than scanners, which may be another factor that explains their better performance. Using a smaller UFOV (2.6° of visual angle), Rubin et al. (2015) found that average coverage for lung cancer screening was only 26.7%. Consistent with research using 2D images, higher coverage was associated with reduced sensitivity. In fact, they estimate it would have taken almost 12 min per case for the images to be thoroughly searched, but average search time was closer to 3 min. In a direct comparison between 2D and volumetric image search, coverage was higher for mammography than breast tomosynthesis over a wide range of UFOV estimates (Aizenman et al., 2017). In fact, overall coverage was less than 30% in volumetric images using the highest UFOV estimate (5°). A similar finding of decreased volumetric image coverage was observed in a study comparing DBT to single-slice DBT (Lago et al., 2018).

**Fig. 5:**
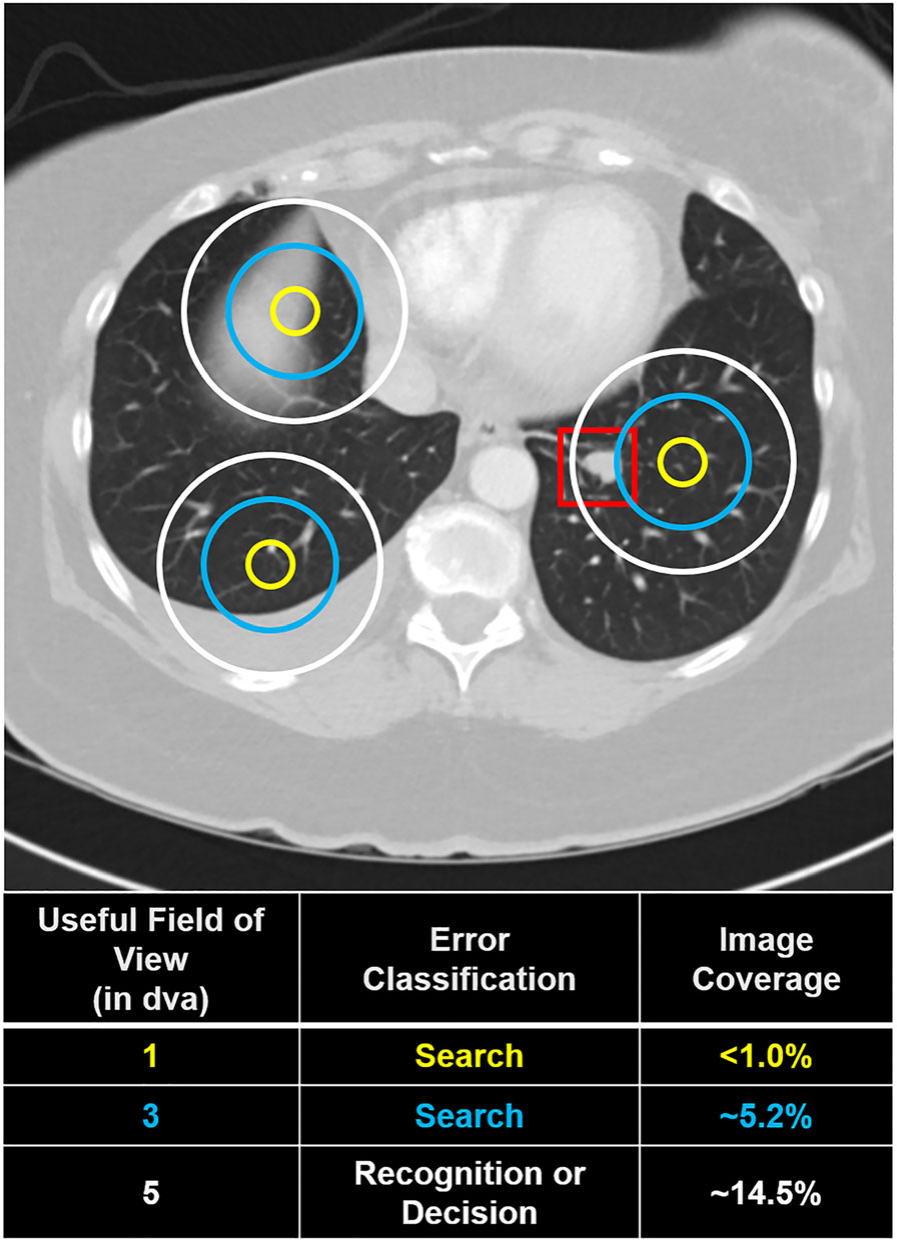
How useful field of view (UFOV) is defined (in terms of degrees of visual angle (dva)) directly influences the outcome of downstream analyses, such as error classification and image coverage. In this hypothetical example, an observer fixated three times (represented by concentric circles) on an image with a visible lung-nodule (located in the red box) but failed to report it. Using a smaller UFOV, the missed abnormality would be considered a search error. However, it would be classified as a recognition or decision error using the largest UFOV estimate. Similarly, estimated image coverage varies considerably with UFOV size. Critically, the size of the UFOV is both task-dependent and observer-dependent (Drew, Boettcher & Wolfe 2017; Young & Hulleman, 2013; Ebner et al., 2017)

Although it is clear that coverage is low in volumetric images, it is impossible to obtain a precise estimate of coverage without an accurate UFOV estimate (Fig. 5). In chest radiography, 5° is a common estimate of UFOV because the vast majority of lung nodules can be detected within that window (Kundel, Nodine, Thickman, & Toto, 1987). However, UFOV is known to decrease with image complexity and task difficulty (Drew, Boettcher & Wolfe 2017; Young & Hulleman, 2013), and research suggests this estimate may be too generous for lung cancer screening in chest CT (Rubin et al., 2015). It is also important to note that UFOV varies substantially with nodule size, image complexity, and reader (Ebner et al., 2017). In future research, it may be necessary to empirically validate UFOV estimates for a particular task or to report results for a range of UFOV estimates (e.g., Aizenman et al., 2017). For example, Rubin et al. (2015) calculated UFOV based on the distance of nodules from central fixation at the moment of recognition, and found that 99.8% of detected nodules were 50 pixels or less from central fixation (2.6° window). Notably, 25% of missed nodules were never within UFOV, which highlights the potential consequences of poor image coverage in large volumetric images. In addition, it is important to understand how UFOV changes as a function of expertise in order to test models of expertise in volumetric images. For example, if experts are able to detect abnormalities more effectively using parafoveal vision, they should have a wider UFOV than novices.

In light of the poor image coverage associated with volumetric images, holistic processing might play a downsized role relative to overall image coverage: it is obvious there could be no behavioral benefit if the slices of the image that contain the abnormality are never visible. For example, there may be a behavioral cost of expertise if there are unexpected abnormalities in an image. We know that contextual cueing is detrimental to performance if the target is not in the expected location (Makovski & Jiang, 2010). Similarly, in medical images, initially incorrect holistic decisions are rarely reverted (Mello-Thoms, 2009) and clinical history significantly affects how images are interpreted (Norman, Brooks, Coblentz, & Babcook, 1992). In volumetric images, these effects may be exacerbated by the increased need to reduce the overall search area and the ability to scroll directly to regions of interest. For example, the most logical way for a radiologist to evaluate a patient for gallstones is to focus on the layers of the image where the gallbladder is present. However, this approach may lead to negative consequences if there are unexpected abnormalities, such as cancer, that are visible on different slices of the image. Although missed incidental findings are not necessarily an error in the context of the assigned task, the undetected cancer may nonetheless result in negative patient outcomes and/or medical malpractice claims. Considering the potential for incomplete image coverage to result in negative consequences for both the patient and the radiologist, future research on this topic is essential.

Given the limitations of memory in search, it is reasonable to question how attention is guided to new locations at all in volumetric images. Eye-tracking during real-world tasks suggests that humans continually sample their environment for information rather than relying on short-term memory (Ballard, Hayhoe, & Pelz, 1995). In fact, some researchers have suggested that the appearance of mnemonic mechanisms in visual search may be driven largely by search strategies rather than memory for previously searched locations (Peterson, Beck, & Vomela, 2007). Observers often adopt a systematic pattern during orderly visual search tasks and display a bias toward horizontal scans of the scene (Dickinson & Zelinsky, 2007; Findlay & Brown, 2006; Gilchrist & Harvey, 2006). Furthermore, working memory capacity (~ 4 items; Cowan, 2001) is typically assumed to be a limiting factor for the number of previous locations that can be maintained in memory (McCarley, Wang, Kramer, Irwin, & Peterson, 2003). However, some studies have found that observers are less likely to refixate on as many as 12 previous fixations and will report with high confidence whether or not a target appeared at those locations (Dickinson & Zelinsky, 2007; Peterson et al., 2007). Working memory capacity limitations may be overcome by maintaining a coarse representation of the general search path rather than a high-resolution memory of the distractor locations (Dickinson & Zelinsky, 2007; Godwin, Benson, & Drieghe, 2013; Peterson et al., 2007). Notably, random deployments of attention to salient stimuli are faster than volitional deployments of attention, which may explain why observers often fail to engage in a systematic search pattern (Wolfe, Alvarez, & Horowitz, 2000). This research suggests it is often a better strategy to randomly sort through large amounts of visual information quickly rather than perform slow systematic searches of the environment. However, it could reasonably be argued that a systematic strategy should play a larger role in radiology due to the need to prioritize accuracy over speed in medicine. Nonetheless, Kundel et al. (1987) calculated that a systematic search strategy through a chest radiograph would require 500 fixations and 3 min of searching, which far exceeds what is typically observed in these tasks (e.g., 1 min, 50 s by expert observers in Christensen et al., 1981). Thus, it seems that radiologists often adopt search strategies that prioritize efficiency over an exhaustive search.

Nonetheless, the sheer size of volumetric images may necessitate some degree of systematic search through the depth of the image in order to counteract a limited memory system. In support of this proposal, Solman and Kingstone (2017) found that partitioning a search array encouraged a more systematic search strategy and led to improvements in explicit recall for previous target locations. Similarly, expert dermatologists exhibited fewer refixations that were separated further in time and were less likely to retrace a scan path than novices (Vaidyanathan, Pelz, Alm, Shi, & Haake, 2014). In addition, there is compelling evidence in other areas of radiology that using a more structured approach might generally help offset memory demands and improve performance. For example, relative to free-form dictation templates, structured templates improve dictation quality (Marcal et al., 2015; Marcovici & Taylor, 2014; Schwartz, Panicek, Berk, & Hricak, 2011), encourage adherence to best practices (Kahn Jr., Heilbrun, & Applegate, 2013), and improve diagnostic accuracy (Bink et al., 2018; Lin, Powell, & Kagetsu, 2014; Rosskopf et al., 2015; Wildman-Tobriner et al., 2017). Similarly, using a checklist with anatomical structures and frequently missed diagnoses improved diagnostic performance in a group of medical students (Kok, Abed, & Robben, 2017, though see Berbaum, Franken Jr., Caldwell, & Schwartz, 2006). In addition, radiologists tend to look at their dictation screens more often following an interruption, presumably in order to remember where they have already searched (Drew, Williams, Aldred, Heilbrun, & Minoshima, 2018). Together, this evidence suggests that interventions that target memory limitations are a worthwhile endeavor, but it is not yet known if adopting a systematic search strategy might also help counteract these limitations in volumetric search.

### How do radiologists decide to terminate search in large volumetric images?

Another challenge for our limited memory in visual search is determining when to stop searching and move on to the next task. In some cases, the answer is simple. If you are looking for honeycrisp apples in a new grocery store, you will stop searching once you have found them. However, how do you know when to stop searching if the store does not sell these apples? In radiology, the problem becomes even more complex; the targets are often unspecified in both appearance and quantity. If the radiologist finds a tumor, there may still be other tumors located elsewhere. The most conservative approach would be to search every relevant pixel of the image. However, time-constraints likely prohibit such a strategy, particularly in light of the increase in the size and number of images generated by volumetric imaging techniques in recent years (McDonald et al., 2015). Furthermore, even if the radiologist has an unlimited amount of time to conduct such a search, a failure to find an abnormality does not mean that an abnormality is not there. In fact, many abnormalities in radiology are fixated on but never reported (Kundel et al., 1978). So how does the radiologist decide when to terminate search given all of this uncertainty?

A model of search termination has been proposed that is similar to a drift diffusion model (Wolfe, 2012). During search, information is acquired about how long or how many items you have searched until a termination threshold is reached. This threshold can be pushed around by variables in your environment, such as the likelihood of a target being present or the reward associated with finding the target. Recently, it has been proposed that search termination may mimic foraging behavior observed in the wild (Cain, Vul, Clark, & Mitroff, 2012; Wolfe, 2013). When an animal forages for food, such as berries, energy intake is maximized by moving on to the next bush when the intake falls below the average intake for that environment, which is known as optimal foraging theory (Charnov, 1976). Observers seem to follow the predictions of this model when searching for multiple targets, and collect items in runs of one target at a time when searching for multiple different types of targets (Cain et al., 2012; Wolfe, 2013; Wolfe, Aizenman, Boettcher, & Cain, 2016). However, it is less clear how quitting behavior changes when the goal is not to collect a large number of abundant, obvious targets but instead to find rare, hard-to-find targets.

Although terminating search too early may lead to negative consequences in radiology, the factors that determine when a radiologist decides to terminate search are poorly understood. Existing models of medical image interpretation focus on what might be considered the front-end of the clinician’s ultimate task of accurate diagnosis: initial perception (Drew et al., 2013; Nodine & Kundel, 1987; Swensson, 1980). It is important to note that these models do little to account for the decision-making that follows perception. This is in contrast to a number of cognitive models, such as a class of drift-diffusion models, that were explicitly designed to account for differences in how long it takes for an observer to reach a decision (e.g., Ratcliff & McKoon, 2008). None of the current models of medical image perception address how a clinician ultimately decides when to stop examining a case. However, it is clear that most true positives are identified very early during search (Berbaum et al., 1991; Christensen et al., 1981; Nodine, Mello-Thoms, Kundel, & Weinstein, 2002) and large portions of CT scans are never searched at all (e.g., Drew et al., 2013; Rubin et al., 2015). As search continues, the likelihood of false positives increases dramatically. This topic is particularly important in the context of volumetric images, which typically take much longer to evaluate than 2D images. Under these circumstances, ability to efficiently move on from a healthy patient’s scans may be a critical indicator of expertise that would be missed by the existing models. For example, in 2D images, experienced radiologists appear to terminate their search when they are still identifying more true positives than false positives, but novices continue until false positives are the dominant response (Christensen et al., 1981; Nodine et al., 2002). Evaluating the time-course of errors with expertise in volumetric imaging is completely uncharted territory and will likely be a fruitful area for future research. In addition, future research could seek to apply a drift diffusion modeling technique to determine how different factors, such as overall workload or the experience of the observer, influence the quitting threshold in volumetric image search.

### How do motor and perceptual processes interact in the evaluation of volumetric images?

Unlike the feature-based searches that are common in the literature on visual attention, search in the real world often involves navigating through large 3D spaces for objects that may be obscured by other objects. When searching a field for targets, participants spontaneously adopt a systematic search path (Riggs et al., 2017). Similarly, when searching for evidence of a crime, participant dyads engage in a highly systematic search and frequently double check their work (Riggs et al., 2018). However, others have found that revisits are rare in real-world visual search, which is attributed to the extra effort required to retrace your steps in locomotive tasks (Gilchrist, North, & Hood, 2001; Smith et al., 2008). Together, this research has profound implications for volumetric image search, which involves both motor and perceptual components as the observer scrolls through depth. In recent years, there has been a growing interest in how motor processes influence visual search when target items must be located by moving other items, termed “manually assisted search.” In some cases, manually assisted search has replicated findings from the visual search literature (e.g., “the low prevalence effect”, Solman, Hickey, & Smilek, 2014). In other cases, new sources of error have been identified. For example, Solman, Cheyne, and Smilek (2012) created an “unpacking” paradigm where the observer could move overlapping virtual items using the computer mouse. The researchers found that target items were often picked up and discarded without being recognized, which suggests that perception and action can be decoupled in visual search. In other words, a decision for action (“discard the selected item”) can precede a decision for identification (“the selected item is my target”). The authors propose that naturalistic visual search engages a perceptual search process that supervises, but does not direct, the motor “unpacking” process (Solman, Wu, Cheyne, & Smilek, 2013).

In radiology, the findings could mean that the motor system decides to move through depth before an abnormality can be identified on the current slice. Although verbal instructions to slow down motor movements were ineffective, forcing the participant to slow down significantly reduced unpacking errors (Solman et al., 2013). At present, it is unknown how the speed of scrolling through the depth of a volumetric medical image relates to diagnostic accuracy or whether these “decoupling” errors occur in radiology. Presenting CT scans at different frame rates has led to mixed outcomes, ranging from no accuracy differences (Bertram et al., 2013) to poorer performance at faster speeds (Bertram et al., 2016). Scrolling speed may also provide insight on the effectiveness of motion onset cues or the development of global processing ability in volumetric imaging. If experts are able to extract relevant information from the images more quickly than novices, they might be less vulnerable to the potentially negative effects of scrolling more quickly, such as “decoupling” errors. In a lung cancer screening task, there were no observed differences in speed between radiologists and naïve observers (Diaz, Schmidt, Verdun, & Bochud, 2015). On average, nodules were detected at a speed between 25 and 30 frames per second (fps). However, it is notable that performance in this task was at ceiling for both experts and novices, and there were no differences in performance across groups. Therefore, it remains possible that differences in scrolling behavior are more important when there is greater variability in performance. Bertram et al. (2013) also found no expertise-related differences in performance when observers looked for a variety of abnormalities in abdominal CT scans presented as fixed-speed videos at 7, 14, or 28 fps. In contrast, Bertram et al. (2016) observed better performance at 5 fps than 3 fps, and experts were better able to adapt to the increased presentation rate in abdominal CT scans. However, both of these studies relied on fixed-speed videos rather than allowing the observers to control their own speed, which limits ecological validity. Clearly, there is not yet a complete picture of how scrolling speed influences search performance in volumetric images. In future research, it may also be beneficial to analyze the distribution of errors as a function of naturalistic scrolling speed. Moreover, it is important to consider the properties of the abnormality itself. Scrolling speed might be an important predictor of abnormality detection  when abnormalities elicit motion onset cues or for smaller abnormalities that are visible on fewer layers of depth. In contrast, it is less likely that scrolling speed predicts the detectability of diffuse or large abnormalities that are visible throughout many slices.

## Concluding remarks and future directions

This review of the literature highlights the many contributions made by researchers toward better understanding volumetric image interpretation. However, due to the contemporary nature of much of this research, much of our knowledge is driven by the data rather than grounded in theory. Although exploratory analyses often lead to important predictions for future research, there is a limit to what can be learned from simply characterizing search behavior. Much like research on 2D medical image interpretation, this approach has revealed substantial variability between observers and experience levels in volumetric image search. However, contrary to the 2D medical image perception literature, models of expertise have not yet been well-established for volumetric image interpretation. In fact, it is unknown how even some of the most ubiquitous findings from the literature, such as increased global processing ability with expertise, apply to volumetric image search. In addition, relatively few expertise studies have been conducted using volumetric images while allowing the observer to freely scroll through depth, which leaves substantial unanswered questions about how scrolling behavior might relate to task performance or develop with experience. Given the increasing popularity of volumetric imaging and the recent Food and Drug Administration (FDA) approval of both breast tomosynthesis and digital pathology, this represents a significant opportunity for researchers interested in helping clinicians understand how to best examine these complex images.

The challenge for the field going forward will be to transition from describing search behavior in volumetric images to establishing models of expertise with testable predictions**.** Ultimately, these models should be able to account for the stimulus, the task, and the observer. Fortunately, researchers are well-situated to make this transition. Almost 50 years of research into medical image perception can be leveraged to make predictions about expertise in volumetric images. This research has led to models of expertise that are associated with a number of well-established eye-tracking metrics, and research has demonstrated it is feasible to adapt these measures to volumetric images (e.g., Helbren et al., 2014, 2015). Furthermore, there are many examples in the literature that highlight the promise of using basic science to make better predictions about medical image perception (e.g., Corbett & Munneke, 2018; Drew et al., 2013; Evans, Birdwell, & Wolfe, 2013), and there is substantial untapped potential for using this approach in volumetric imaging as well.

A basic science approach may help the field transition from a more descriptive, computational analysis of what radiologists do to a better understanding of *how* the radiologists perform their task, which represents another level of analysis in our understanding of complex systems (Marr, 1982). As outlined in this review, there are many relevant findings from basic science that are untested in volumetric medical images. Some of the most exciting avenues for future research may be determining the limitations of memory in volumetric image search, the features that capture attention in volumetric images (e.g., motion onset cues), or the interactions between motor and perceptual processes when scrolling through depth. By grounding future research firmly in the literature on basic science and medical image perception, the field is poised to make substantial progress in our understanding of volumetric image search in the coming years.

## Data Availability

Not applicable.

## Abbreviations

2D: Two-dimensional
3D: Three-dimensional
CT: Computed tomography
DBT: Digital breast tomosynthesis
FIT: Feature integration theory
FPS: Frames per second
IOR: Inhibition of return
MRI: Magnetic resonance imaging
ms: Millisecond
ROI: Region of interest
UFOV: Useful field of view
